# Electrolysis in Cancer of Breast

**Published:** 1873-11

**Authors:** 


Cancer of Female Breast and Electrolysis.—Dr. Crosby exhibited a specimen of cancer of the female breast, removed the afternoon of the meeting by him from a lady aged —. She had been for a long time under the electrolytic treatment, but without benefit. The clinical interest in this case was centred in the application of electrolysis to the surface of the wound, in the hope that any remaing germs of disease might be eradicated at the same time that the healing process might be hastened. He had made it a practice in his late operations to make use of the process, and thought that good had come of it.

